# A small subset of protected areas are a highly significant source of carbon emissions

**DOI:** 10.1038/srep41902

**Published:** 2017-02-10

**Authors:** Murray B. Collins, Edward T. A. Mitchard

**Affiliations:** 1 School of GeoSciences, University of Edinburgh, The King’s Buildings, Alexander Crum Brown Road, Edinburgh EH9 3FF, UK.

## Abstract

Protected areas (PAs) aim to protect multiple ecosystem services. However, not all are well protected. For the first time, using published carbon and forest loss maps, we estimate carbon emissions in large forest PAs in tropical countries (N = 2018). We found 36 ± 16 Pg C stored in PA trees, representing 14.5% of all tropical forest biomass carbon. However the PAs lost forest at a mean rate of 0.18% yr^−1^ from 2000–2012. Lower protection status areas experienced higher forest losses (e.g. 0.39% yr^−1^ in IUCN cat III), yet even highest status areas lost 0.13% yr^−1^ (IUCN Cat I). Emissions were not evenly distributed: 80% of emissions derived from 8.3% of PAs (112 ± 49.5 Tg CO_2_ yr^−1^; n = 171). Unsurprisingly the largest emissions derived from PAs that started with the greatest total forest area; accounting for starting forest area and relating that to carbon lost using a linear model (r^2^ = 0.41), we found 1.1% outlying PAs (residuals >2σ; N = 23), representing 1.3% of the total PA forest area, yet causing 27.3% of all PA emissions. These results suggest PAs have been a successful means of protecting biomass carbon, yet a subset causing a disproportionately high share of emissions should be an urgent priority for management interventions.

Forests provide multiple services to humans, including climate regulation, nutrient cycling, and stable supplies of fresh water^1^. As such, managing them well is vital for human well-being and the continued functioning of the global ecosystem and economy. Nonetheless natural forest was lost at the rate of 10.6 × 10^6^ ha yr^−1^ during the 1990 s and 6.5 × 10^6^ ha yr^−1^ between 2010 and 2015^2^. This has been driven by the rapid expansion of industrial agriculture and plantations to meet increasing global demand for commodities, and often exacerbated by the spread of fires in remaining forests which have been degraded by selective logging and other activities^3^.

These processes cause biodiversity loss^4^, and contribute to climate change: forest loss accounts for approximately 18% of all anthropogenic carbon emissions, increasing atmospheric greenhouse gas concentrations and thereby changing the global climate^5^. Therefore, forest loss simultaneously reduces the availability of Earth’s carbon sinks whilst increasing emissions. The topic is thus a central issue in environmental management and is of enormous concern to environmental scientists, policy makers, the general public, and those private sector actors who are implicated in driving deforestation, such as companies developing oil palm plantations^3^.

Accordingly, there are a range of public policy responses that aim to reduce forest loss. One of the most common policy tools used is the establishment of protected areas (hereafter ‘PAs’), which include national parks and other forms of protection in which extractive activity or other anthropogenic disturbance is either illegal or highly regulated. These PAs are created under national law and the protection that applies is therefore highly variable. However PAs are also categorised by an international body, the International Union for the Conservation of Nature (IUCN) according to their management regime and protection status, from Ia (the highest level of protection) through to VI (the lowest level of protection; see Table 1)^6^. One of the most important international agreements underpinning the establishment of protected areas is The United Nations Convention on Biological Diversity^7^. This recommends that 10% of each country be set aside for protected areas for the conservation of biodiversity. The World Database on Protected Areas (WDPA) records 111,897 such areas on land^8^, covering 15.4% of the world’s land area.

Whilst the global establishment of PAs is encouraging, it is clear that ostensibly protected forest areas continue to experience deforestation^9^,^10^. Indeed, data describing continuing deforestation both inside and outside protected areas^11^,^12^,^13^,^14^ combined with the acknowledgement that it is essential to include forest management in any solution to climate change, catalysed the United Nations Framework Convention on Climate Change (UNFCCC) to create a mechanism called ‘Reducing Emissions from Deforestation and Degradation in developing countries, and the sustainable management, conservation and enhancement of forest carbon stocks’ (REDD+). This is an umbrella strategy designed, in part, to provide incentives for forest rich countries to contribute to climate change mitigation by reducing forest loss rates, and hence emissions. REDD+ is being implemented in so-called developing countries, more specifically under the United Nations Framework Convention on Climate Change (UNFCCC) treaties, the ‘Non-Annex I Parties to the UNFCCC’ (hereafter ‘NA1’). These countries include almost all the world’s tropical forests, in addition to a little non-tropical forest within countries such as China and Chile.

At a global level REDD+ is still at a nascent stage, with participating countries running a series of preparatory ‘readiness’ activities. This includes developing sufficient satellite remote sensing capacity to be able to demonstrate that the country in question is indeed reducing deforestation levels; and assessing the most nationally-appropriate policies to implement REDD+. However some countries are more advanced in their engagement with REDD+. One outstanding example is Indonesia, whose government negotiated an agreement with the Government of Norway in 2010 to reduce deforestation in return for $1bn of finance over seven years^15^. This deal led directly to revised forest management policy, specifically the issuance of a nationwide logging moratorium as a means to reduce carbon emissions from forest. Yet this is only one of a range of policy options that forest countries can legitimately explore under REDD+. As the definition in the previous paragraph makes clear, the support of forest conservation is an activity that can be employed in order to reduce forest loss and receive REDD+ finance. This may involve the support of existing protected areas which are experiencing deforestation despite their legally protected status. In this case, an important question arises over which of these areas are experiencing the most rapid rates of forest loss, and are thus priorities for management interventions.

Physically, the areas that provide the greatest cost effectiveness of mitigation are those with a combination of both high forest carbon stocks (large areas of high biomass, intact forests), and also high rates of forest loss. The former (high stocks) are likely to be found in tropical forests, but it is hoped that the latter (high forest loss rates) are rare due to existing conservation measures; it is thus unclear whether PAs should form a priority for REDD+. At the global level, the carbon stocks in protected areas, and the emissions arising from forest loss from within them, remain unquantified. Identifying these areas could help improve the impact of protected area management interventions, for instance under REDD+, and thereby improve the efficiency of climate change mitigation action given a budget constraint.

As such we were motivated to assess the status of NA1 PAs. First, we wanted to quantify the total terrestrial carbon stock stored within them, to understand existing PA’s importance in regulating the global climate now, and to explore whether the highest levels of protection were being attributed to the most carbon rich intact forest areas. Then, given the already widespread knowledge that protected forests are experiencing forest loss^10^,^11^,^12^, we wanted to quantify for the first time the carbon impact of gross forest losses in absolute terms. In undertaking this analysis, we aimed to reveal the potential climate change impact of ineffective policy implementation through failing protected area enforcement. Finally, by accounting for the original conditions of the protected areas (their original forest cover in 2000), we sought to identify those protected areas where forest loss and carbon emissions were disproportionately large. In doing so our aim was to reveal a subset of PAs where intervention under REDD+ could provide the greatest marginal mitigation benefit, without having to legislate for change in land use designation. Formally, our hypotheses were that: 1. Forest carbon density is higher in high status protected areas; 2. Forest loss rates are inversely proportional to IUCN protection status. 3. Total forest loss, and hence total carbon emissions, are directly proportional to starting forest area across PAs.

## Results: Summary findings

### Carbon stocks

**Figure 1:**
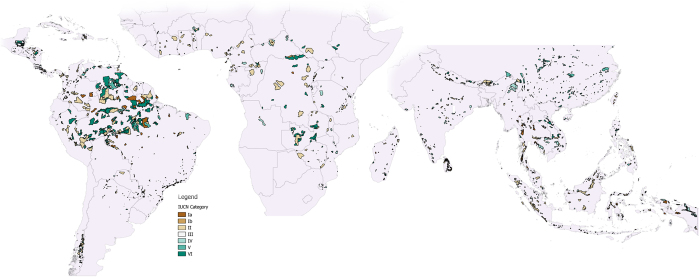
Map of the 2018 PAs assessed in Non-Annex I countries, colour coded by IUCN categories I-VI. IUCN Shapefiles downloaded from www.protectedplanet.net^8^. Map created by the authors using QGIS 2.10.1 Pisa. http://www.qgis.org/and GIMP 2.8.14 https://www.gimp.org/.

We found that PAs are biased overall slightly towards higher biomass ecosystems: the 2,018 large (>10 km^2^), NA1, >50% tree cover (in the year 2000) PAs in our dataset (Fig. 1) contained 35.8 ± 15.7 Pg C (28.0 ± 13.7 Pg C in aboveground biomass, AGB; 7.8 ± 2.0 Pg C in below ground biomass, BGB). This is 14.5% of the total biomass C estimated to be held in tropical countries as estimated from the same carbon stock dataset^16^. These C stocks exhibit non-linear spatial distribution across PAs: 80% of stocks are stored in only 11% (n = 213) of the PAs. This is in part because the size of reserves varies by nearly four orders of magnitude, from 10 km^2^ (*Araras*, Brazil) to 51,335 km^2^ (*Sur del Estado Bolivar*, Venezuela); in part because some PAs contained only 50% forest cover (our minimum threshold for a given PA’s forest cover to be included in the dataset) whereas many had 100%, and finally because mean carbon stocks varied from 6.8 to 189.3 Mg C ha^−1^.

### Forest loss

**Figure 2:**
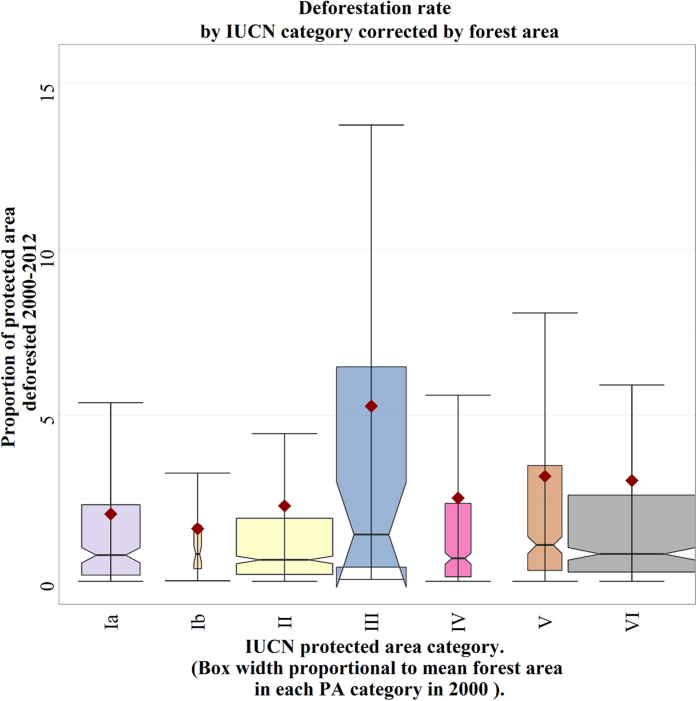
Forest loss rates in IUCN category protected areas 2000–2012. Each box shows the middle half of the data, bounded by the 1^st^ and 3^rd^ quartiles with the median the central notched line in between, and the whiskers 1.5 times the inter-quartile range. The heavy skew in the data is indicated by the mean values (red diamond) being far higher than the median values in each class.

Mean forest loss rates were 0.18% yr^−1^ across all PAs. Whilst this does not seem extreme, it is higher than would be expected given that no IUCN PA should be subject to any forest clearance (Table 1, only Category VI are allowed any extractive activities, and these should be ‘low-level, non-industrial’; Category VI are not outliers in our dataset, see Fig. 2, so it is not the extraction rates in some of these that are skewing our overall deforestation rate estimates). In total, between 2000 and 2012 forest loss across the PAs caused losses of 461** ± **202 Tg C; or 38 ± 17 Tg C yr^−1^. In comparison, total annual tropical deforestation emissions have been estimated at 89–461 Tg C yr^−1^  ref. 5, and 570–1,222 Tg C yr^−1^ ref. 17. Fluxes from PAs are thus non-negligible, and given that PAs also have significant additional ecological and cultural values, the case for better protection is far stronger than these carbon-only figures suggest.

### Significance of Brazil and Indonesia for absolute gross emissions from Pas

Two countries are outstanding for their absolute gross forest losses and emissions from protected areas: Brazil and Indonesia. Brazil was the largest source of gross emissions from protected areas, which is not surprising as it has the largest total protected area network. However it is more concerning that Indonesia is the second largest source of gross PA emissions, as it has the third largest of area under protection of any country, and its PAs only cover 15% of the area of those in Brazil, yet it produced 25% of Brazil’s emissions. Equally, while there is evidence of deforestation rates slowing in Brazil, they appear to be increasing more generally throughout SE Asia^13^. No major tropical forest country avoided the problem (see SI): in total 248 PAs across 32 countries lost over 1 Tg CO_2_ from their PAs in the 21^st^ century. Clearly the improved management of PAs should be assessed by all countries as they prepare their commitments on emissions reductions under the UNFCCC.

### Significance of Cambodia for disproportionate amount of total protected forest carbon lost

Accounting for the amount of carbon stored in protected area forests, a different pattern emerges: Cambodia stands out uniquely as having lost a remarkable 16.5% of its protected forest carbon since 2000. This was followed by Guatemala (9.4%); Mozambique (8.1%); Côte D’Ivoire (8.0%); and Grenada (6.7%; all from only one PA, *Grand Etang*). For full list of proportional protected carbon losses per country, see SI).

### The top gross emitting PAs

**Figure 3:**
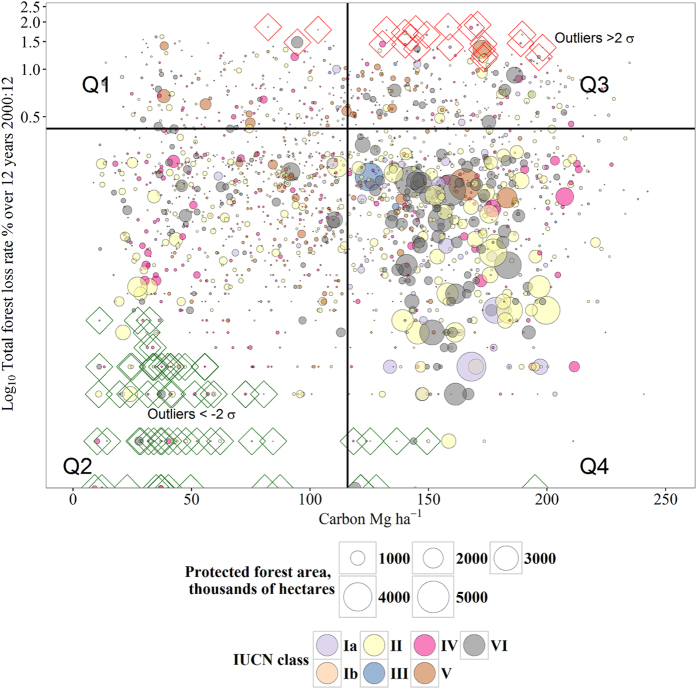
Gross deforestation rates and mean carbon stocks ha^−1^ in 2,018 PAs, with each bubble representing an individual protected area scaled by its original forest area in 2000; thereby characterising 21^st^ century status and trends in PAs in UNFCCC Non-Annex I countries. The vertical axis is the Log_10_ of deforestation rates 2000–2012 per site, corrected for original forest cover, and the horizontal axis indicating the mean forest carbon stocks. The image is bisected vertically by the mean forest carbon in the sample (115.6 Mg C ha^−1^), and horizontally by the mean of forest loss rates 2000–12 (Log_10_2.6% = 0.41), producing four quadrants. PAs in Q1 have high gross forest loss rates, but low carbon stocks, hence large impacts for biodiversity and other ecosystem services, but low carbon emissions. PAs in Q2 have both low forest loss rates and carbon stocks, hence emissions are low. The majority of the low-emission outliers are found here, highlighted in green (n = 69). PAs in Q3 have high carbon stocks, but high loss rates, hence are large sources of emissions. The majority of the high-emission outliers are found here, highlighted in red (n = 23). PAs in Q4 have high carbon stocks but low forest loss rates. These are the world’s intact high-biomass forests serving as carbon stores and sinks.

The data suggest that the bulk of high carbon stock PAs have been well-protected (most points are in Quadrant 4 of Fig. 3). However a small proportion dictate most of the losses. The distribution of forest loss rates are highly positively skewed, with means much higher than medians (Fig. 2); and emissions from only 8.5% (n = 171) of the PAs caused 80% (112 ± 49.5 Tg CO_2_ yr^−1^) of the total. This is a significant quantity of carbon to come from 171 PAs: in comparison the UK’s entire transport sector was 116.7 Tg CO_2_ in 2013^18^. Overall, approximately one third (32%) of these high-emission PAs are in Brazil; with a further 13% in Indonesia. Hence these two countries, manage almost half (45%) of all the highest gross emitting IUCN-categorised PAs. Remarkably, a third (33.1%) of gross emissions derived from only 10 individual PAs. The top five gross emitting sites were: 1. *Triunfo do Xingu,* Brazil (IUCN V; 13.6% total emissions); 2. *Floresta Nacional do Jamanxim*, Brazil (IUCN VI; 4.5% total emissions); 3. *Maya biosphere buffer zone*, Guatemala (IUCN VI; 4.0% total emissions); 4. *Patuca* National Park, Honduras (IUCN II; 2.2% total emissions); and 5. *Sebangau,* Indonesia (IUCN II; 1.8% total emissions).

## Results: Robustness checks

### Validation of forest loss

**Figure 4:**
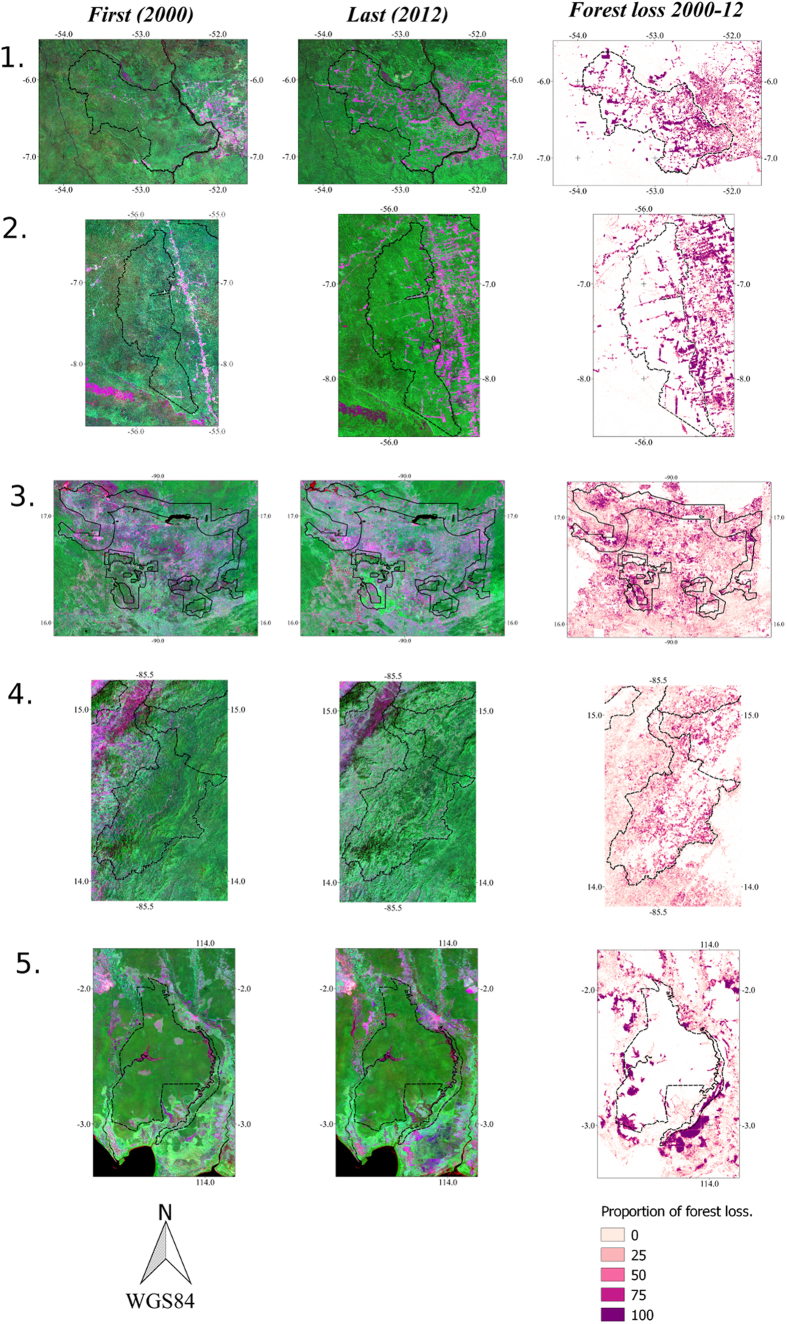
The top five emitting IUCN PAs between 2000 and 2012: 1. *Triunfo do Xingu*, Brazil (IUCN V), 2. *Floresta Nacional do Jamanxim*, Brazil (IUCN VI); 3. *Maya biosphere buffer zone*, Guatemala (IUCN VI); 4. *Patuca* National Park, Honduras (IUCN II); 5. *Sebangau,* Indonesia (IUCN II). For each of these numbered PAs we show below from left to right the first (from 2000) and last (from 2012) images from the global forest loss dataset^13^, followed by an image of the forest loss estimated between these periods^13^. We aggregated the change image to 300 m, with each pixel value indicating the proportion of forest lost 2000–2012. Image created using GIMP 2.8, https://www.gimp.org/. Source: Hansen/UMD/Google/USGS/NASA. Licence details: http://earthenginepartners.appspot.com/science-2013-global-forest/download_v1.2.html.

We sought to independently verify that such large changes were indeed occurring, focussing on the five sites above with the highest gross emissions. First we present the Landsat 7 image composites produced for ~2000 and ~2012^13^ for the top five PAs with the highest gross emissions, as shown in in Fig. 4 alongside the forest losses estimated to have occurred between 2000 and 2012. This served as a visual verification from the raw datasets that such huge forest losses were occurring within the borders of the protected areas. Second, we present evidence from the literature of severe environmental degradation in these five areas:

1. ***Triunfo Do Xingu, Brazil** “ongoing and planned dams, road paving, logging and mining, together with increasing demand for agricultural commodities, [and] continued degradation of upper headwaters*^19^*”; and “Triunfo do Xingu and Serra do Pardo correspond with crisis areas despite being designated PAs*^20^”.
2. ***Floresta Nacional do Jamanxim, Brazil*** Decree 258 introduced in 2009: a proposal to degazette (the complete removal of legal protection status) 1.3 × 10^6^ ha of this forest^21^. However at the time of our analysis the WDPA database still lists this site as having 1.3 × 10^6^ ha under protection, from which area we calculated emissions.
3. ***Maya Biosphere Reserve, Guatemala**“[i]n the Multiple-Use Zone of Guatemala’s Maya Biosphere Reserve, the usufruct rights to timber and non-timber forest resources were granted through concession agreements to 12 community organizations and two private timber companies in the late 1990* *s and early 2000* *s. After more than a decade, some concessions are successfully managing forests for multiple uses while others have had limited success or failed completely*^22^*”.*
4. ***Patuca, Honduras** “In spite of these efforts (*creation of PAs; deforestation observation prior to study period)*, colonization of state forest lands has proceeded unabated*^23^”; and *“(l) and clearing along the Patuca and Wampu rivers threatens to fragment the contiguous Platano, Tawahka and Patuca reserves*^24^”.
5. ***Sebangau, Indonesia** (i) llegal logging and deforestation are currently reducing the forested area*^25^”.

## Results: Hypothesis testing

### ‘Forest carbon density is higher in high status protected areas’

The highest priority Category Ia PAs had the highest average carbon density (149.5 Mg C ha^−1^), suggesting high protection status is on average awarded to more intact or simply higher biomass forest. Yet the second highest category of protection, Ib forests, had a mean of 87.5 Mg C ha^−1^, which is less than the lowest level of protection category VI (117.4 Mg C ha^−1^; see Table 1 for density per PA category). Since the highest status forests in Ia do indeed have the highest carbon density, we do not reject our first hypothesis. Nonetheless, excluding category Ia, there is less of a clear distinction between the other remaining categories. This suggests that there is a relationship between the very highest category protection and the degree to which the forest area concerned remains (or started) as high biomass or intact forest. However without time-series data of carbon storage we cannot assess causality.

### ‘Forest loss rates, and hence emissions are inversely proportional to IUCN protection status’

According to our hypothesis the highest status PAs (Ia) should have the highest protection and hence experience lower rates of forest loss than the lower status areas (II-VI). However, contrary to our expectations, Category Ia PAs experienced forest loss rates of 0.17% yr^−1^ over the study period (2.03% total loss 2000–12, Fig. 2). This is concerning: Category Ia are meant to have very limited human access, but it is clear that the incentives for exploitation must outweigh any disincentives given by additional legal protection. The highest rates of forest loss were in the category III PAs, which experienced losses of 0.44% yr^−1^ and the losses were lowest in category II PAs, at 0.13% yr^−1^. Therefore we reject our second hypothesis.

### ‘Forest loss, and hence carbon emissions, are directly proportional to the original forest area of tropical protected areas’

The gross estimates described in the summary results, do not account for the size of PAs. We used a regression model to account for this, with the log of carbon emissions dependent upon the log of the forest area of each PA in the WDPA database (r^2^ = 0.41). The model revealed 23 positive outliers, emitting more carbon than would be expected for their size, defined as those observations with studentised residuals >2σ (the areas shown outlined in red in Fig. 3 and shown in Table 2). These 23 protected areas constitute only 1.1% of the protected areas sampled, and represent together only 1.3% of the total forest area in the sample, yet represented 27.3% of all protected area emissions. Conversely, we identified 3.4% of the sample (N = 69) as outliers producing significantly fewer emissions than would be expected for their surface area (studentised model residuals <−2σ; the observations shown outlined in green in Fig. 3). These protected areas represented 1.4% of the total forest area of the sample, yet caused only 4 × 10^−3^% of emissions. Given this non-linearity, we rejected our third hypothesis, though the regression model does show that there is a general trend to increasing emissions with starting forest area.

## Discussion

We have provided the first estimate of carbon emissions from deforestation in protected areas across Non-Annex I countries, finding significant carbon emissions based on estimates of gross forest loss in the 21^st^ century. The losses are unevenly distributed, with 10 sites contributing a third of all emissions and one site in Brazil contributing 13.6% of the total carbon emissions. We verified the largest absolute forest losses from PAs by drawing upon field observations published in the literature. These were ascribed variously to mismanagement, illegal logging, fire and the expansion of agriculture within protected area borders, despite that legal status. This is disturbing since despite the high forest loss statistics reported across the globe, a sense of environmental security may be provided by the knowledge of the existence of a global network of protect areas: a sense which we have verified is false. Crucially, we have quantified for the first time the climate impact of this misperception, with a total of 32 countries losing over 1 Tg CO_2_ from at least one of their PAs in the 21^st^ century. This finding indicates that the continued protection of PAs should be assessed by all countries as they consider their commitments on emissions reductions under the UNFCCC.

On a country-by-country basis, and in terms of absolute volume of emissions, it is perhaps not surprising to find Brazil at the top of the list since it has a huge estate of protected high carbon stock forest. By comparison the relatively larger contribution from Indonesia is more concerning, illustrating the continuing problems the country has managing its protected areas. These findings present both a danger and opportunity for policy makers: both Brazil and Indonesia have received big investments aimed at reducing their rates of forest loss, most dramatically the pledges from the Norwegian government to both countries, but also significant capacity building and direct support from multilateral organisations. We have illustrated that as this additional funding is supplied to these two countries to reduce emissions from deforestation, the conservation of existing PAs should not be neglected as a central activity. However, on a proportional basis, it is actually Cambodia that is outstanding, having lost a remarkable 16.5% of its protected forest carbon in only 12 years, suggesting it deserves a larger international profile as the epicentre of PA forest loss.

On the level of individual protected areas, and in addition to absolute forest loss calculations, we discovered that emissions from PAs were not proportional to their original forest areas: 1.1% of the sample, representing only 1.3% of the forest area we studied, caused over a quarter of all emissions from protected areas. In other words these 23 sites had about twenty times greater emissions than would be expected given their starting forest size when considering the whole dataset.

We undertook the analysis on the basis that the WDPA database was accurate and up to date. However, at least some of the forest losses we observed from the ostensibly protected forests listed therein may have been taking place as a consequence of changes in legal status. Indeed, protected areas are being downgraded, downsized and de-gazetted globally^21^,^26^. Yet whilst this may provide an explanation at some sites for the processes that we have quantified, substantively it means that that protected forests are being lost now through a combination of legal and illegal means.

Ultimately, many of the study countries are experiencing high population growth and rapid economic development, placing their forest resources under increasing pressure. Discount rates are typically high in NA1 economies, which means that any money received today is worth more than money tomorrow. In addition, high returns to land use options like palm oil plantation development mean that converting forest land to other uses is likely to be far more financially attractive than conservation. Further, these two factors may interact: for instance large undiscounted returns can be obtained for the conversion of forest to oil palm today, whilst promised REDD + funds may be obtained at a discounted rate tomorrow.

Our analysis quantifies the impact of such land use change pressures in PAs in terms of carbon emissions. Whilst the literature shows that enforcing these PAs is not simple^10^,^11^,^12^, the skewed spatial distribution of both carbon and forest loss indicate that large climate change mitigation benefits could be achieved in the forest sector through intervening at those sites which are both under legal protection and causing disproportionate carbon emissions, by targeting the outliers that we have presented here.

## Methods

We calculated forest biomass carbon stocks and losses in PAs across UNFCCC Non-Annex I (‘NA1’) countries by using a combination of maps 1) global forest loss^13^ (raster, 30 m resolution, 2000–2012); 2) IUCN PA polygons^8^ (vector) for all terrestrial NA1 designated and proposed PAs categories I-VI; 3) forest carbon stocks^16^ (raster, 1 km resolution, dated early 2000 s) and 4) Land cover^27^ (raster, 1 km resolution), a ‘widely accepted forest classification scheme^28^’ that has been assessed to be 95% accurate^29^.

There were 5,628 NA1 PAs in total across the tropics, covering a combined 6.2 million km^2^, about 11% of the total land area of those countries we examined. Since we were dealing with pixels of 1 km^2^ resolution for the original land cover and the biomass map, we were concerned about potential errors arising from analysing those very small PAs whose GIS area recorded in the WDPA database was of the same order of magnitude as the land cover classification pixel size, hence we only assessed those PAs of over 10 km^2^ (removing N = 1744 observations, but just 0.07% of the total area). We then removed any area with fewer than ten x 1 km^2^ pixels classed as forest on the same basis (N = 816). We further restricted the database by focussing on PAs which were designated to conserve forest, upon the basis that a majority of their land area would be covered in forest. In practice we did this by removing any PAs whose recorded total GIS area contained <50% forest pixels (N = 1049) in the Global Land Cover 2000 database, which is a harmonised land cover product for the world referenced to the year 2000^27^. This left us with 2018 observations, which represented 2.58** ± **0.129** **m km^2^ of forest under some degree of protection (Fig. 1).

We resampled the coarse resolution raster datasets to a common 300 m grid using nearest neighbour resampling for carbon and land cover datasets. This means that the underlying values were not changed. We then used area averaging for the forest loss dataset, which resulted in a raster wherein pixel values were the proportion of a 300 m pixel lost 2000–2012, using IDL (Harris). We chose 300 m as a compromise between minimising errors due to pixels overlapping the edges of PA polygons, and computational efficiency. We performed calculations with Zonal Statistics in QGIS 2.14.0^30^. First we corrected the forest loss data to reflect the fact that forest loss can only have occurred in pixels that were originally covered with forest. We calculated this using a multiplier derived from the ratio of pixels in classes 1–8 (forested) in 2000 to the total GIS area of the PA recorded in the WDPA database. We assumed carbon is 50% of total biomass stocks. Hence we calculated total carbon as: area of forested GLC2000 pixels within each PA x (mean PA AGB ha^−1^ + BGB ha^−1^) × 0.5; and carbon losses by multiplying total carbon stocks by the corrected proportion of forest loss. We produced charts using R/GGplot2^31^,^32^. We also calculated the carbon loss statistics using the uncorrected forest loss as a sensitivity analysis.

There are errors and uncertainties associated with any estimate of forest loss, biomass and of carbon emissions. Here they derive from misclassification errors in the landcover classification dataset, and model errors in the biomass maps of above and below-ground biomass. The landcover classification is estimated to be 95% accurate^29^, hence we applied 5% errors to estimates of above and below ground biomass, and the total amount of forest under consideration, on the basis that misclassification errors would be distributed across the entire dataset. To be conservative, for the biomass map data, we used the maximum relative error in the continuous error layer provided with the biomass map of 43.9% for AGB; and 21% for BGB^16^. We calculated the absolute values of the AGB and BGB biomass estimates and added these to estimate total biomass estimates. We included BGB in our estimates of carbon loss on the basis that following forest loss, associated BGB is committed to loss following precedents in the literature^33^. We did not include soil carbon losses, as the timescale and proportion of loss following clearing is much more uncertain, but that absence means that our estimates of losses are bound to be underestimates.

### Sensitivity analysis using uncorrected forest loss data

The resolution of our carbon stock data (1 km) is much lower than our forest loss data (30 m). Therefore in the main analysis we performed a correction for the carbon stocks of PAs with less than 100% forest cover, assuming that the carbon stocks and losses were both concentrated in the forested portion of the PA. In order to confirm that this correction was not unduly changing the results or introducing an artefact, we also performed the calculations with carbon stocks uncorrected for the proportion of a PA that is forest-covered. Since the minimum threshold for forest area in our analysis was 50%, the maximum correction factor possible was a multiple of two. However, the correction that we ultimately applied was in general far smaller: overall C losses are 12% higher using the corrected data, with the total C losses from PAs using the corrected data being 38.4 Pg ± 16.9 Pg C, compared to 34.2 Pg C ± 15.0 Pg C using the uncorrected data. The full results are provided for comparison in the SI.

## Regression modelling

**Figure 5:**
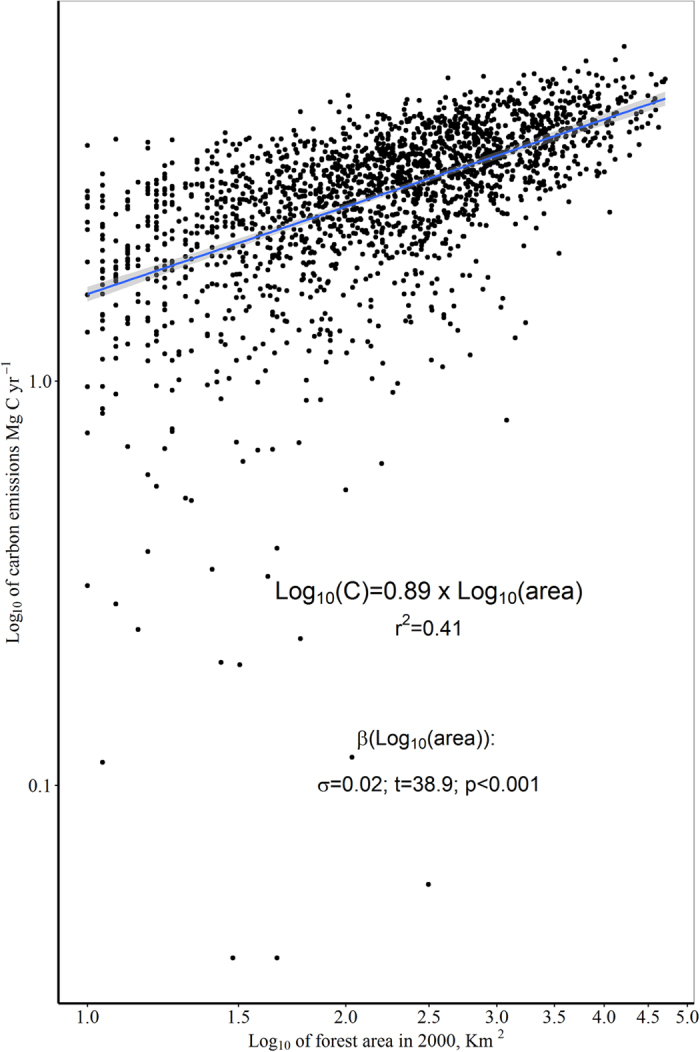
Regression model used in the estimation of outliers. Log_10_ forest area explains almost half of the variation in Log_10_ of carbon emissions. This model allowed us to identify those sites which are disproportionately producing carbon emissions, given their original forest cover in the year 2000.

In order to determine which observations were statistical outliers we created a regression model in R^31^ with estimated carbon emissions as the dependent variable, and the forest cover in the year 2000 area (from the Global Land Cover^27^ database) as the independent variable. We defined outliers as those observations with studentised residuals of >2σ; with positive values being areas producing disproportionately more emissions than expected, and the negative values showing fewer emissions than expected for the given forest area (Figs 3 and 5).

## Additional Information

**How to cite this article:** Collins, M. B. and Mitchard, E. T. A. A small subset of protected areas are a highly significant source of carbon emissions. *Sci. Rep.* **7**, 41902; doi: 10.1038/srep41902 (2017).

**Publisher's note:** Springer Nature remains neutral with regard to jurisdictional claims in published maps and institutional affiliations.

## Supplementary Material

Supplementary Information

## Figures and Tables

**Table 1:** IUCN PA categories and characteristics.

IUCN Category	Description^11^	Mean Carbon density across reported area of PA; Mg C ha^−1^	number of PAs
Summary data in the right hand columns refers to the subset of PAs which we examined, with >50% forest cover in the year 2000, and larger than 10 km^2^, N = 2018.			
**Ia. Strict Nature Reserve.**	*‘Category Ia are strictly protected areas set aside to protect biodiversity and also possibly geological/geomorphical features, where human visitation, use and impacts are strictly controlled and limited to ensure protection of the conservation values’.*	149.5	207
**Ib Wilderness Area**	*‘Category Ib protected areas are usually large unmodified or slightly modified areas, retaining their natural character and influence without permanent or significant human habitation, which are protected and managed so as to preserve their natural condition’.*	87.5	69
**II National Park**	*‘Category II protected areas are large natural or near natural areas set aside to protect large-scale ecological processes, along with the complement of species and ecosystems characteristic of the area, which also provide a foundation for environmentally and culturally compatible, spiritual, scientific, educational, recreational, and visitor opportunities’.*	117.5	552
**III Natural Monument or Feature**	*‘Category III protected areas are set aside to protect a specific natural monument, which can be a landform, sea mount, submarine cavern, geological feature such as a cave or even a living feature such as an ancient grove. They are generally quite small protected areas and often have high visitor value’.*	116.0	36
**IV Habitat/Species Management Area**	*‘Category IV protected areas aim to protect particular species or habitats and management reflects this priority. Many Category IV protected areas will need regular, active interventions to address the requirements of particular species or to maintain habitats, but this is not a requirement of the category’.*	101.9	413
**V Protected Landscape**	*‘A protected area where the interaction of people and nature over time has produced an area of distinct character with significant, ecological, biological, cultural and scenic value: and where safeguarding the integrity of this interaction is vital to protecting and sustaining the area and its associated nature conservation and other values’.*	113.3	357
**VI Protected area with sustainable use of natural resources**	*‘Category VI protected areas conserve ecosystems and habitats together with associated cultural values and traditional natural resource management systems. They are generally large, with most of the area in a natural condition, where a proportion is under sustainable natural resource management and where low-level non-industrial use of natural resources compatible with nature conservation is seen as one of the main aims of the area’.*	117.4	384

**Table 2:** Protected areas producing higher emissions than expected given their original forest area in 2000 (defined as observations >2σ; Ν =23).

Name	Mean above ground biomass ABG Mg ha^^−1^	Country	Forest area in 2000, Km^2^	IUCN Cat	Hansen corrected forest loss rate % 2000–2012	Total Carbon stored (AGB + BGB) Mg C	Carbon lost Mg C yr^−1^	Model residuals (standardised as studentised residuals, calculated with respect to std. deviation of the model residuals)
Snoul	266.7	Cambodia	103	IV	82.9	1,757,877	121,376	2.8
Sultan Thaha Syaifuddin	247.2	Indonesia	83	VI	73.4	1,313,292	80,331	2.6
Sungai Dumai	128.6	Indonesia	18	V	72.0	148,087	8,881	2.2
Snoul	262.3	Cambodia	428	IV	69.5	7,185,222	415,977	2.7
Periquito	225.9	Brazil	12	VI	63.4	173,512	9,164	2.4
Aguateca	161.5	Guatemala	15	II	60.7	155,041	7,838	2.2
Araras	206.6	Brazil	10	VI	60.1	132,225	6,622	2.3
Tesso Nilo	218.7	Indonesia	784	II	51.3	10,975,521	468,845	2.5
Bukit Tiban	296.1	Malaysia	59	II	46.4	1,118,123	43,192	2.5
Mutum	233.1	Brazil	105	VI	45.5	1,566,521	59,342	2.4
Mandor	222.5	Indonesia	28	III	39.1	398,765	12,997	2.2
Maya	147.9	Guatemala	6472	VI	30.4	61,240,359	1,551,570	2.1
Bangkiriang	295.0	Indonesia	92	IV	29.2	1,736,702	42,244	2.3
Phnom Kulen	270.5	Cambodia	228	II	28.4	3,946,778	93,536	2.2
Beng Per	203.9	Cambodia	1671	IV	28.0	21,810,104	508,706	2.2
Bien Lac-Nui Ong	218.9	Vietnam	223	IV	27.4	3,124,029	71,431	2.1
Distrito Regional De Manejo Integrado Del Rio Minero	248.6	Colombia	273	VI	24.2	4,342,716	87,442	2.1
Milian-Labau	309.52	Malaysia	15	Ia	23.7	297,140	5,858	2.0
Jaci-Paraní	226.74	Brazil	2067	VI	22.6	29,995,347	564,732	2.2
Triunfo do Xingu	269.63	Brazil	16677	V	21.8	287,787,642	5,226,470	2.3
Montanas Mayas Chiquibul	271.4	Guatemala	623	VI	16.9	10,822,222	151,949	2.0
Samlaut	307.1	Cambodia	411	VI	15.7	8,077,181	105,896	2.0
Patuca	270.7	Honduras	3943	II	14.9	68,306,435	845,501	2.1

These are priority targets for management interventions e.g. under REDD+. The PA in Cambodia called ‘*Snoul*’ appears as two separate areas in the IUCN WDPA database.
